# N‑Substituted Fluorinated Polybenzimidazoles: An Easy Post-Modification Strategy for Improved CO_2_ Separation

**DOI:** 10.1021/acsapm.5c03888

**Published:** 2026-03-05

**Authors:** C. Aguilar-Lugo, M. Rojas-Rodriguez, A. E. Lozano, L. Fomina, S. Fomine, L. Alexandrova

**Affiliations:** † Instituto de Investigaciones en Materiales. Universidad Nacional Autónoma de México, Circuito Exterior, Ciudad Universitaria, Coyoacán, 04510, Ciudad de México, México; ‡ Facultad de Química, Universidad Nacional Autónoma de México, Circuito Exterior, Ciudad Universitaria, Coyoacán, 04510, Ciudad de México, México; § Instituto de Ciencia y Tecnología de Polímeros, ICTP-CSIC, Juan de la Cierva 3, 28006, Madrid, Spain

**Keywords:** Polybenzimidazole, CO_2_ Separation, N-Substituted PBIs, Gas Separation Membrane, Post-Polymerization Modification, Fluorinated PBI, Thermal Decomposition, Tertiary Amine Groups

## Abstract

This study details synthesizing and characterizing a series of N-substituted aromatic polybenzimidazoles (N-PBIs) obtained via post-polymerization modifications of fluorinated polybenzimidazole (PBI-6F). The modifications involved the introduction at imidazole nitrogen of aliphatic chains bearing tertiary amine groups, affording *N*,*N*-dimethylethylamine and *N*,*N*-dimethylpropylamine N-substituted PBIs, PBI-DMEA, and PBI-DMPA, respectively. Their properties were compared with the performance of N-substituted PBI bearing a pure propyl chain, without an amine group in the end (PBI-Pr). All N-PBIs demonstrated enhanced processability, exhibiting solubility not only in dipolar aprotic solvents but also in such ordinary solvents as chloroform and THF. Their performance as gas separation membranes was tested and showed that the membranes with amine groups in the substituent had enhanced permselectivity for the CO_2_/CH_4_ gas pair, which was rather explained by an increase in the solubility parameter of CO_2_. However, the presence of the aliphatic amines also resulted in an unusually low decomposition temperature for these polymers. The mechanism of thermal decomposition and solubility and diffusivity parameters were confirmed by corresponding calculations.

## Graphical abstract

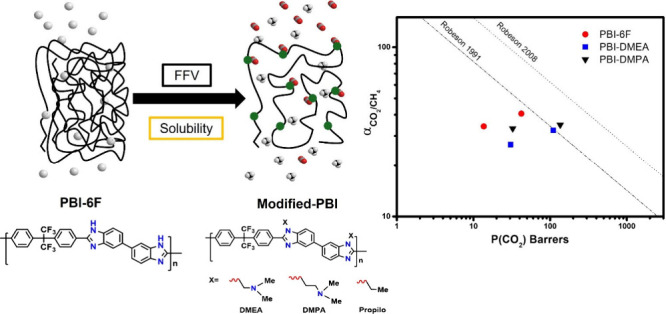

## 1 Introduction

Membrane gas separation technology is a powerful tool for solving global environmental problems, such as the greenhouse effect, natural gas sweetening, etc.^1–3^ Gas separation using polymeric membranes is a growing business that offers competitive cost and simplicity in operation and has important industrial applications, including the production of high-purity nitrogen, gas dehydration, removal of acid gases, and recovery of hydrogen from process streams. Despite thousands of new polymers that have been evaluated as potential gas separation membranes for the last 20 years, the same group of polymers, such as aromatic polysulfones or polyimides, dominates the market because they have a better balance between permselectivity and cost.^3–5^ Therefore, it is still an important task to find a simple way to modify traditional polymers to improve their gas transport properties.

The polymeric membranes based on aromatic polybenzimidazoles (PBIs) have attracted attention due to their high intrinsic H_2_/CO_2_ separation capability.^6–11^ Besides, PBIs are known for their excellent chemical and thermal stability and suffer less from aging and plasticization, which are the main drawbacks of polyimide and polysulfone membranes. However, the rigidity of PBIs’ structure makes them poorly soluble in most organic solvents, which complicates the formation of a membrane from these polymers. The most studied PBI so far is poly­(2,2′-*m*-phenylene-5,5′-bibenzimidazole), *m*-PBI, the only commercialized PBI (Celazole).^6,9,11^ *m*-PBI is a low-permeable material due to the limited free volume produced by its tight chain packing, which restricts appreciable transport at room temperature, but several attempts were made to improve its permselectivity. For example, hollow fiber membranes were obtained through a multistep post-spinning process, exhibiting permselectivity values with a H_2_ permeance of 500 GPU and H_2_/CO_2_ selectivities of 19 at temperatures above 250 °C.^9^ The H_2_/CO_2_ selectivity of *m*-PBI was significantly enhanced by chemical cross-linking of the polymer in the solid state.^10^ The permeability was affected to a lesser degree, which allowed for the improvement of whole permselectivity at 200 °C. However, this also resulted in the formation of an insoluble gel fraction.

Various approaches were tried to enhance the processability and gas permeability of PBIs, such as blending with other polymers,^12,13^ PBI composites,^14^ and post-polymerization N-substitution.^15,16^

Thus, various N-substituted organosilane PBIs were obtained by deprotonation of Celazole, which were much more soluble in organic solvents than the parent *m*-PBI.^15^ Although the yields were not high enough, this opened the possibility for improving some properties. Consequently, a systematic study of a series of polymers that were N-modified by alkyl groups derived from two parent PBIs, differing by the acid fragments, was carried out with a focus on their gas transport properties.^16,17^ N-Substituted PBIs were obtained in quantitative yields, demonstrating lower viscosity and much better solubility due to the elimination of intermolecular hydrogen bonding and looser chain packing. It also resulted in significantly higher permeability for all gases, but the effect was more pronounced in the polymers derived from the denser parent PBI (*m*-PBI). As expected, the bulkiest substituent resulted in the largest density decrease and the largest permeability increase. The effect on selectivity was more complex and dependent on the gas pairs. The substitution was predominantly effective in the variation of gas diffusivity rather than in their sorption in the polymer matrix. On the other hand, a significant increase in the sorption of CO_2_ was noted in the cases of aliphatic substituents. In contrast, the sorption coefficients of other gases slightly decreased, which led to an increase in the CO_2_-based selectivities.

Influence of the main chain structure on the physical and gas transport properties of PBIs was also investigated,^6–8,18^ and, as expected, the inclusion of bulky hexafluoroisopropylidene (6F) fragment into the polymer backbone resulted in much more soluble and permeable polymers,^18^ demonstrating better permselectivity at 35–45 °C than *m*-PBI.^8^ Additionally, it was easier to form dense self-supported membranes from PBI-6F.

Synthesis of PBI-6F readily occurs from 3,4′-diaminobenzidine (DAB) and 4,4′-(hexafluoro­isopropylidene) bis­(benzoic acid) (HFA), affording a linear, high molecular weight, soluble in organic solvents polymer practically without any gel fraction using the appropriate reaction conditions.^19,20^ However, the influence of post-polymerization modification of PBI-6F on the physical and gas transport properties has not yet been reported.

This work describes the post-polymerization modification of the N–H reactive sites of PBI-6F with two dimethylamine aliphatic chains of different lengths. According to the reported data, the addition of the amine moiety not only enhances the polymer’s processability but also increases the permselectivity of CO_2_.^16,21,22^

## 2 Experimental Section

### 2.1 Materials

All reagents, 3,3′-diaminobenzidine (99%), DAB, 4,4′-(hexafluoroisopropylidene) bis­(benzoic acid) (98%), HFA, sodium hydride dry (90%) NaH, 1-methyl-2-pyrrolidone anhydrous (99.5%), NMP, N,N-dimethylacetamide (99%), DMAc, 2-chloro-*N*,*N*′-dimethylethylamine hydrochloride (99%), Cl-DMEA, 3-chloro-*N*,*N*′-dimethylpropylamine hydrochloride (99%), Cl-DMPA, 1-bromopropane, Br-Pr, chloroform (≥99.8%), methanesulfonic acid, MSA, phosphorus pentoxide, P_2_O_5_, and sodium bicarbonate, NaHCO_3_, were purchase from Sigma-Aldrich.

### 2.2 Synthesis of PBI-6F

Polymer (PBI-6F) was prepared by direct polycondensation between DAB and HFA in Eaton’s reagent (ER) according to the literature.^23^ Typically, in a Schlenk flask with a mechanical stirrer, N_2_ inlet, and CaCl_2_ drying tube, DAB and HFA were added in an equimolar quantity, the solids were dissolved at room temperature in ER, and the mixture was heated in an oil bath at 180 °C for 1 h to form a viscous solution. The polymer obtained was precipitated in water and neutralized with NaHCO_3_. It was purified by dissolving in NMP and precipitating in water 3 times. Finally, it was washed with methanol and dried at 80 °C under vacuum to a constant weight.

### 2.3 N-Substitution Methodology

The chemical modification of PBI-6F was carried out as described below. In a two-neck round-bottom flask, 1.5 g of PBI-6F was dissolved in 20 mL of an 80:20 (v/v) NMP:DMAc mixture at room temperature. Then, 5 equiv to every PBI-6F repeat unit of the corresponding alkyl halide (Cl-DMEA, 2.02 g; Cl-DMPA, 2.20 g; Br-Pr, 1.73 g) was added under vigorous stirring. The solution was heated to 70 °C, and 1.01 g (42.09 mmol) of NaH was gradually added. The reaction mixture was kept for 5 h at this temperature and then maintained at 45 °C for another 24 h. Finally, the reaction mixture was poured into deionized water, and the modified polymer was extracted with chloroform from the water precipitate. The solvent then was rotary evaporated, and the residue was washed again with deionized water and dried in a vacuum at 80 °C to a constant weight. The yields were about 90%.

### 2.4 Preparation of Membranes

The polymeric membranes were prepared by solution casting using 5% (w/v) solutions in NMP (PBI-6F) and CHCl_3_ (all N-modified polymers) on a flat glass surface. The solutions were filtered prior to casting. In the case of PBI-6F, a step-heating scheme was applied to evaporate NMP. Initially, NMP was evaporated at 60 °C under vacuum for approximately 5 h until it was possible to peel the film off the glass. The film was dried in a vacuum at 80 °C for 1 h and then at 120 °C for another 6 h. In the cases of N-modified PBIs, CHCl_3_ was allowed to evaporate slowly at room temperature, and then the formed solid films were dried at 80 °C for 2 h. Finally, for better removal of the residual solvent, the membranes were heated in a vacuum at 200 °C for 10 h.

### 2.5 Characterizations

^1^H NMR spectra were recorded on a Bruker Advance III HD 400 at 400 MHz. A Bruker Alpha-P infrared spectrometer was used to obtain FT-IR spectra. Inherent viscosities (η_inh_) were determined in 0.5 g/dL NMP solutions using a Cannon Ubbelohde viscometer No. 100 L161 at 30 °C. Thermogravimetric analysis was performed on a TA Instruments Q5000 equipment by the Hi-Res method under nitrogen at a heating rate of 20 °C/min. Wide-angle X-ray scattering, WAXD, measurements were performed on a Bruker D8 Advanced X-ray diffractometer (Ni-filtered Cu–Kα radiation graphite monochromator). The average *d*-spacing was obtained from the Bragg’s equation:

1 nλ=2dsinθ

where *d* is the *d*-spacing, θ is the scattering angle, λ is the wavelength of the incident radiation, and *n* is an integer number related to the Bragg order.

Densities were determined using a Mettler Toledo Advanced MS Semi-Microbalance coupled with a density kit based on Archimedes’ principle. The samples were weighed in air and into a known-density liquid (high-purity isooctane). The measurements were performed at room temperature, and the density was calculated from the following expression:

2 $$\rho_{sample}=\rho_{liquid}\frac{W_{air}-W_{liquid}}{W_{air}}$$

The density data were applied to evaluate chain packing using the fractional free volume (FFV), which was calculated using the following relation:

3 $$FFV=\frac{V_{e}-{1.3V}_{W}}{V_{e}}$$

where *V*_*e*_ is the polymer specific volume and *V*_*W*_ is the van der Waals volume, which was obtained by molecular modeling using the semiempirical method Austin Model 1 (AM1) in Hyperchem version 8.0. Gas permeability and diffusion were determined in a gas permeation cell of constant volume. All gases had purities of >99.99% and were obtained from Praxair Corp. Gas permeability coefficients were determined under steady-state conditions at 3 atm upstream pressure.^24^ The measurements were performed at 30 °C for each pure gas. Permeability values for the polymers were calculated from the slope of downstream pressure vs time curve (*dp*(*t*)/*dt*) when steady state conditions had been achieved, according to the expression

4 $$P=\frac{273}{76}\frac{Vl}{ATp_{o}}\frac{dp\left(t\right)}{dt}$$

where *A* (cm^2^), *V* (cm^3^), and *l* (cm) are respectively the effective area, the downstream volume, and the thickness of the film; *T* is the temperature in K, and *p*_*o*_ (cmHg) is the pressure of the feed gas in the upstream chamber. *P* is usually expressed in Barrer [1 Barrer = 10^–10^(cm^3^(STP)­cm)/(cm^2^ scmHg)]. The ideal selectivity for a gas pair was calculated by taking the ratio of the gas permeability.

5 $$\alpha_{A/B}=\frac{P_{A}}{P_{B}}$$

where *P*_*A*_ and *P*_*B*_ are the permeability coefficients of pure gases A and B, respectively.

The apparent diffusion coefficient (*D*) was obtained by using the time-lag (θ) method by the relationship

6 $$D=\frac{L^{2}}{\theta }$$

where θ is the time-lag. The apparent solubility coefficient (*S*) was calculated from the ratio between the *P* and *D* coefficients:


7 $$S=\frac{P}{D}$$

## 3 Results and Discussion

N-Modified polybenzimidazoles were synthesized via a two-step procedure as shown in Figure 1, where in the first step a high molecular weight with η_inh_ = 1.3 and solubility in dipolar aprotic solvents, such as dimethyl sulfoxide (DMSO), dimethylformamide (DMF), or *N*-methyl-2-pyrrolidinone (NMP), PBI-6F was obtained as described elsewhere.^23^ N-Substitution of PBI-6F with the corresponding alkyl halide proceeded using a strong base to deprotonate nitrogen of the imidazole group and resulted in an approximately 90% yield of N-modified polymers. The modified PBIs were readily soluble not only in dipolar organic solvents but also in THF and chloroform. The inherent viscosity values of N-substituted PBIs were lower than that of pristine PBI-6F (Table 1), which may be a result of reduced intermolecular interactions because of hydrogen bonding elimination.

**Figure 1:**
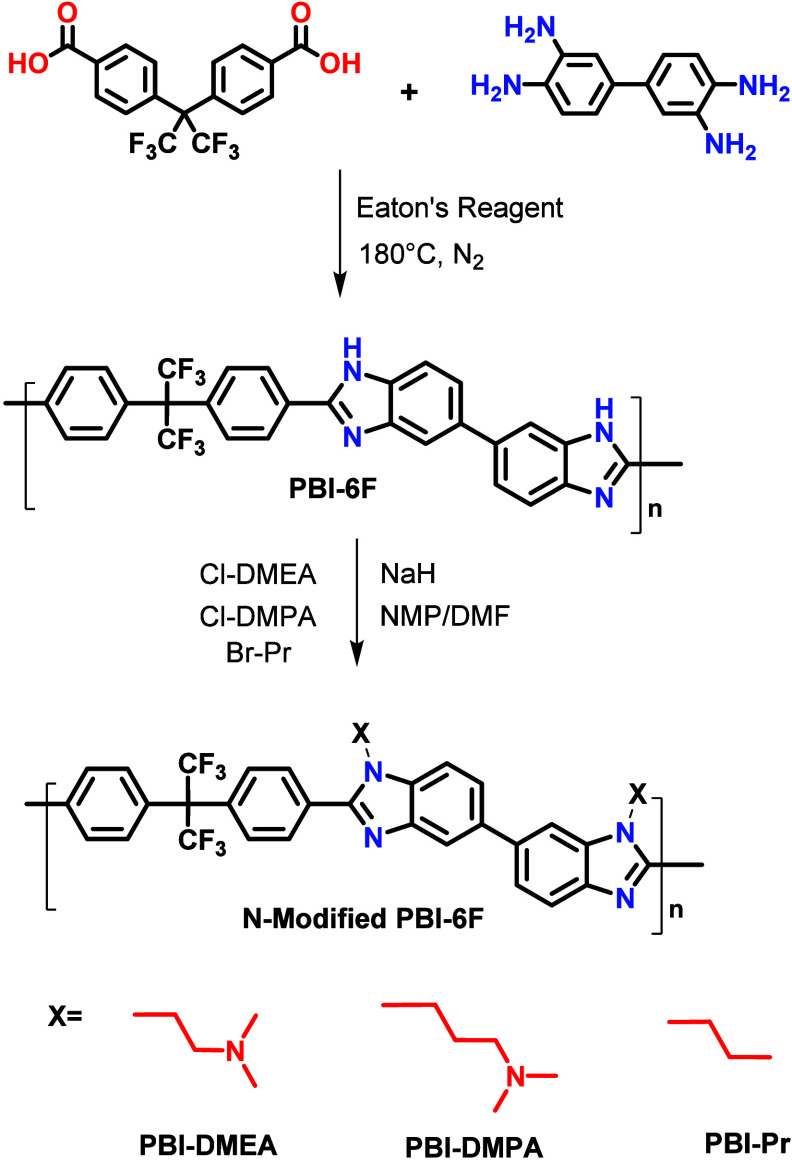
Scheme of the synthesis of PBI-6F and its subsequent modifications.

**Table 1:** Some Physical Properties of Polybenzimidazole Membranes

**Polymer**	***T***_**10%**_ **(°C)**	**η**_**inh**_ **(dL/g)**	**Density** ^a^ **(g/cm** ^**3**^ **)**	***V*_w_ (cm** ^**3**^ **/g)**	**FFV**	***d*** **-spacing (nm)**
**PBI-6F**	548	1.30	1.3871	0.4749	0.1454	0.521
**PBI-DMEA**	424	0.91	1.2842	0.5090	0.1502	0.524
**PBI-DMPA**	402	0.89	1.2674	0.5164	0.1492	0.558
**PBI-Pr**	503	0.85	1.2821	0.5099	0.1501	0.571

a Densities were measured after 300 °C treatment.

The polymers’ structures were confirmed by ^1^H NMR spectroscopy; the spectra are given in Figure 2. The spectrum of pristine PBI-6F was identical to those previously reported for this polymer, showing all typical signals: at 13.2 ppm (2H) corresponding to the proton attached to the imidazole nitrogen (Hc), signals from the aromatic protons were detected in the 8.6–7.6 ppm area.^19,23,25^ Peaks observed between 4 and 2 ppm were due to water and residual solvent. As for modified PBIs, the aromatic protons, Ha, Hb, Hd, Hf, and Hh, were observed between 8.2 and 7.1 ppm, while the signal from the imidazole proton at 13.3–13.2 ppm was not detected anymore. The disappearance of this signal, together with the integration of the peaks from the protons belonging to the adjusted alkyl tails as, for example, for PBI-DMEA: (ppm) 4.3 (4H, He), 2.0 (4H, Hd), and 0.97 (6H, Hc), clearly demonstrated quantitative N-substitution for all polymers.

**Figure 2:**
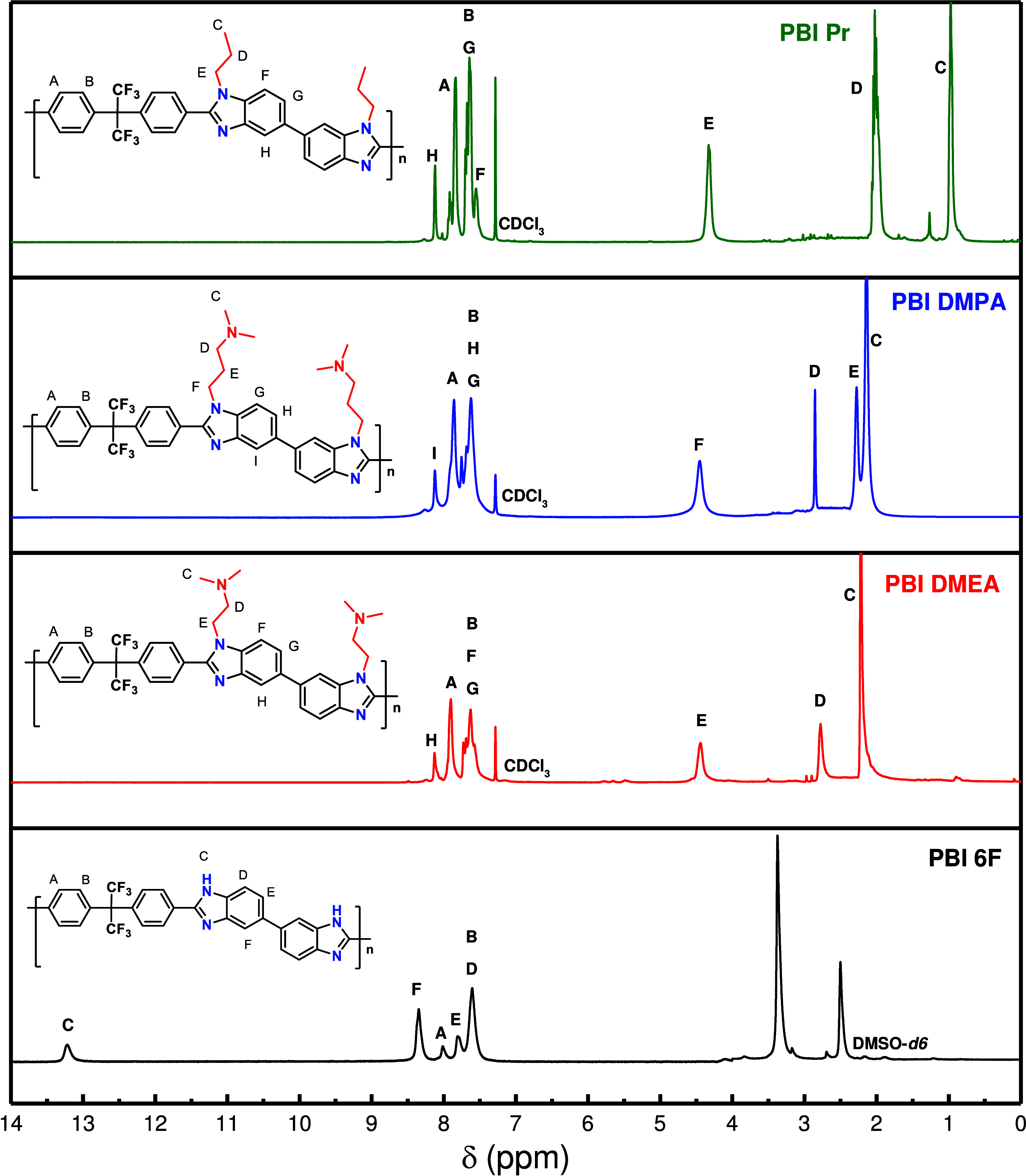
^1^H NMR (400 MHz) spectra of PBI-6F (DMSO-*d*_6_) and N-modified PBIs (CDCl_3_).

All PBIs were also analyzed by ATR-FTIR spectroscopy, and the spectra are presented in Figure 3b–d. Consistently with the reported data,^23,24^ the FTIR spectrum of pristine PBI-6F exhibited a characteristic set of bands, such as a broad one at 3500–2800 cm^–1^ due to stretching vibrations of the bounded −NH···H, C=C, and C=N stretching frequencies typical for a benzimidazole ring in the region of 1650–1380 cm^–1^ and C–F stretching vibrations between 1260 and 1150 cm^–1^. All characteristic absorptions are marked in different colors in Figure 3. The absorptions corresponding to the C=C/C=N benzimidazole vibrations between 1700 and 1350 cm^–1^ remained in the FTIR spectra of the modified PBIs, as well as characteristic C–F stretching vibrations, but the –NH···H band was absent. Instead, the group of C–H aliphatic stretching bands at 2900–2800 cm^–1^ was observed in their spectra. Thus, FTIR data confirmed the successful *N*-alkyl modifications of PBI-6F.

**Figure 3:**
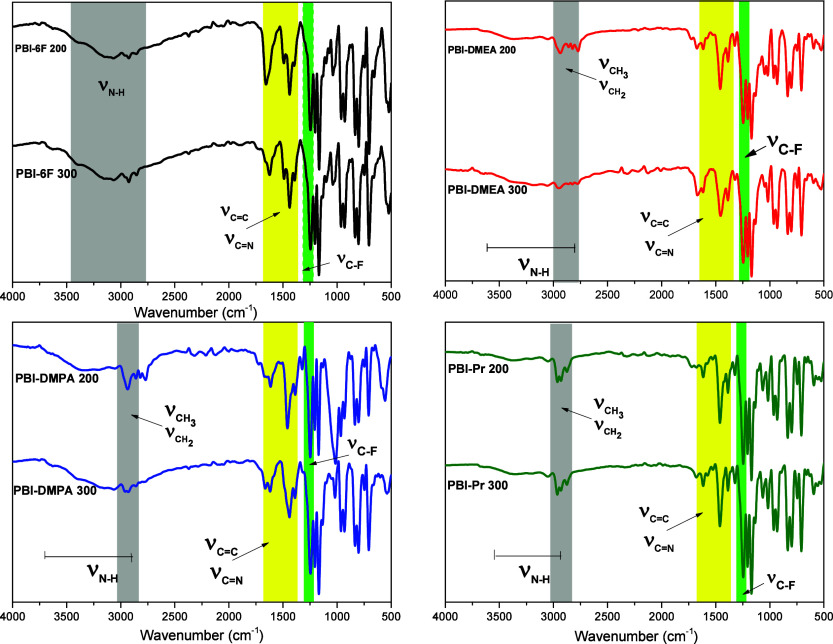
FT-IR spectra of PBI-6F and chemically modified PBIs after thermal treatment at 200 and 300 °C.

Since the goal of the present research was to investigate how such N-modification influenced the membranes’ gas permselectivity, it was important to remove all the residual solvents and absorbed water from the membranes. As described in the Experimental Section, all membranes were subjected to stepwise thermal treatment in a vacuum with heating at 200 °C for 10 h in the final step. However, it was not enough to eliminate completely the liquid trapped within the membranes. The TGA-DTA method was used to verify the presence of the residual liquids in the membranes. It is important to stress that the measurements were conducted on the fabricated membranes and not on the polymer powders. The evolutions of the TGA curves corresponding to PBI-6F and PBI-DMEA membranes after heating at 200 °C for 10 h and additional treatment at 300 °C for 1 h are shown in Figure 4a and b, respectively. Thermograms of other N-substituted membranes were quite similar to that of PBI-DMEA. As one can see, the TGA curves of the membranes after prolonged treatment at 200 °C in a vacuum showed a slight slope starting from 200 °C for PBI-DMEA and from 330 °C for PBI-6F. Such shape indicates the presence of a low molecular weight compound that gradually evaporates during heating. These weight losses often turn out to be residual solvent trapped within the synthesized polymer. The percentage of weight loss was higher for PBI-6F, around 5%, than that for PBI-DMEA, within 2%. As was mentioned above, PBI-6F membranes were cast from high-boiling NMP, while all N-modified membranes were cast from CHCl_3,_ and this may explain the difference in the observed onset temperatures and percentage of weight loss. Although 200 °C is much higher than the CHCl_3_ boiling temperature, according to our experience, sometimes a complete elimination of not high-boiling solvents from the polymer matrix did not occur even at very high temperatures.^26^ To ensure the complete removal of the residual solvents trapped in the polymer matrix, all membranes were subjected to an additional heat treatment: heating at 300 °C for 1 h in an Ar atmosphere. As can be seen for PBI-6F and PBI-DMEA membranes dryed at 300 °C (Figure 4a and b), their TGA curves were horizontal from the beginning, without any gradual inclination until their initial mass loss temperatures. For example, thermograms of the pristine PBI-6F membrane after such treatment looked as expected for this polymer, with the initial mass loss temperature above 500 °C;^19,20^ the small broad exothermic peak around 400 °C seen in the DTA curve before the additional treatment disappeared after heating at 300 °C (Figure 4a). As for thermogram of the PBI-DMEA membrane, even after additional heat treatment, its onset temperature remained very low for aromatic PBI, but the shoulder around 300 °C observed on its DTA curve before treatment at 300 °C disappeared. The TGA-DTA thermograms of all membranes after the corresponding drying process are given in Figure 4c. No drops were observed at the initial stages of heating; all thermograms were horizontal from the beginninig to their initial mass loss temperatures, indicating the complete removal of the solvents in the membranes and evidence that the thermal procedure was adequate. It was important to confirm that the chemical structure of the polymers was not affected by such heat treatment. That is why the FTIR spectra of the membranes were run again after heating at 300 °C (Figure 3). As one could observe, the spectra did not change; all characteristic absorptions discussed above were presented in the after-heating spectra of the membranes, maintaining the same ratio between the bands. So, we concluded that the drying procedure employed did not result in any important structural changes in the membranes but allowed complete removal of all residual substances from them.

**Figure 4:**
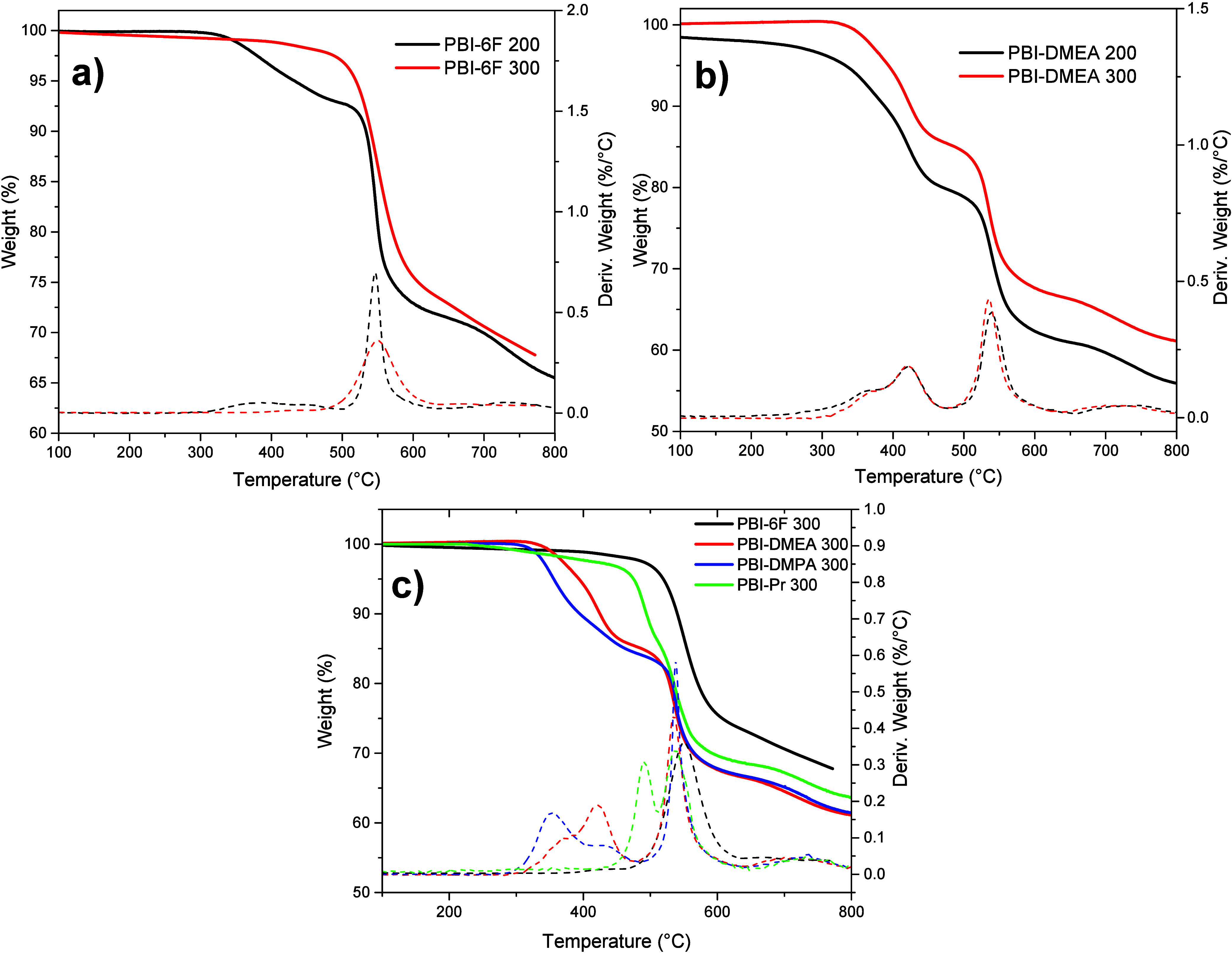
Thermograms of (a) PBI-6F and (b) PBI-DMEA membranes after treatment in a vacuum at 200 °C for 10 h and after additional heating at 300 °C for 1 h in Ar; (c) thermograms of the membranes after their complete drying process at 300 °C.

TGA curves indicate that N-modified PBIs started decomposing at lower temperatures than pristine PBI-6F, which may be expected according to our experience and earlier observations in the literature.^17,19,20,27,28^ Curiously, the difference between the initial decomposition temperatures of PBI-6F and *N*-alkyl-modified PBI-Pr was about 50 °C, which agreed with data reported in the literature for other *N*-alkyl-substituted PBIs,^17,27,28^ while the decompositions of *N*-alkylamine-substituted polymers, PBI-DMEA and PBI-DMPA started just above 300 °C, i.e., almost 200 °C lower than that of PBI-6F. Based on experimental studies, PBIs were supposed to decompose by a radical mechanism, with the first breakdown occurring in the imidazole ring and, in the case of N-substituted PBIs, decomposition started with the scission of the bond between imidazole nitrogen and carbon of the substituent.^27^ Indeed, the DTA curve of PBI-Pr clearly exhibited two exothermic well-separated peaks at 490 and 547 °C, matching the first and second weight loss steps. The first low-temperature weight loss was 13%, which coincided with a loss of a propyl group. PBI-DMEA and PBI-DMPA also exhibited two-step decompositions; their DTA peaks of the second step were in the range of the main weight loss of PBI-6F and PBI-Pr membranes, i.e. 500–600 °C, but their first-step DTA exotherms started at 310 °C and had quite complex shapes. For a deeper understanding the thermal degradation mechanism of these N-substituted PBIs, some theoretical consideration of bonds’ strength in the alkyl tails was performed. Figure 5 shows the calculated bond dissociation energies (in kcal/mol) for two model molecules: **A** with a propyl tail at the imidazole nitrogen and **B** bearing an (*N*,*N*-dimethylamino)­ethyl substituent at the imidazole nitrogen. The calculations were performed using the Gaussian 16 rev. C.01 software package,^29^ employing the robust range-separated, dispersion-corrected hybrid functional ωB97XD^30^ in combination with the large triple-ζ def2-TZVP basis set. The weakest bond in each molecule is highlighted in red. As seen in Figure 5, the weakest bond in molecule **A** is the C–N bond between the imidazole nitrogen and alkyl carbon, which agreed with the above-mentioned experimental studies.^27^ As for molecule **B**, the C–C bond within the alkyl amine substituent was the weakest. Besides, this bond was notably weaker than the weakest C–N bond in compound **A**, which explains the lower temperature degradation of *N*-amino alkyl PBIs in comparison with *N*-alkyl-substituted ones. The differences in bond dissociation energies also account for the other features in the thermal degradation behavior of the N-substituted PBIs detected by TGA. In the case of PBI-Pr, the propyl group was cleaved in a single step from the polymer backbone, as the weakest bond was the one connecting the propyl group to the polymer chain, confirmed by a unimodal and symmetrical shape of the first DTA peak. In contrast, N-amino alkyl substituted PBIs lost their substituents in two steps: initially via cleavage of the weakest C–C bond, followed by the loss of the rest of the substituent. Indeed, DTA peaks of the low-temperature weight loss observed for PBI-DMEA and PBI-DMPA were not unimodal. They may be deconvoluted into at least two peaks, indicating a complex process occurring in their low-temperature decomposition steps.

**Figure 5:**
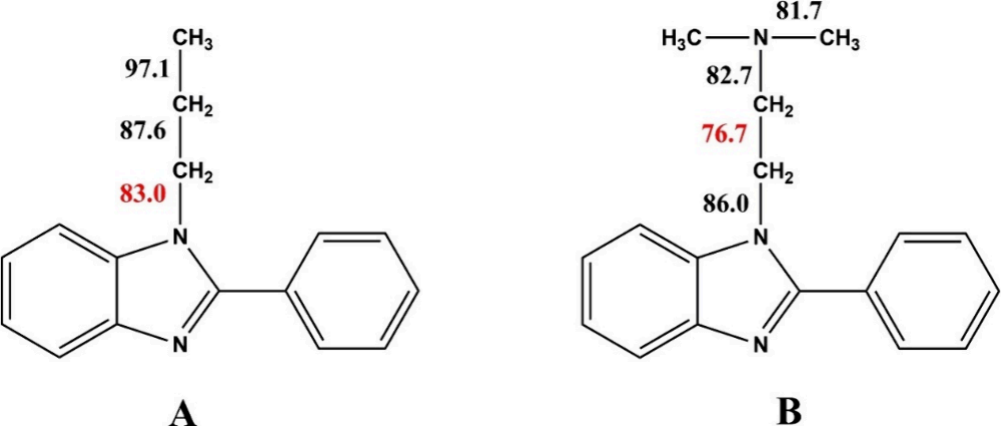
Calculated bond dissociation energies (kcal/mol) for model molecules (A) PBI-Pr and (B) PBI-DMEA. Weakest bonds are highlighted in red.

Chain packing density is one of the key parameters for the determination of gas permeability for polymer membranes. Wide-angle X-ray diffraction (WAXD) and experimental measurement of the membranes’ density were used to evaluate their chain packing. The results obtained are displayed in Table 1, and WAXD patterns are given in Figure 6. All polymers were completely amorphous, showing typical diffuse halos.

**Figure 6:**
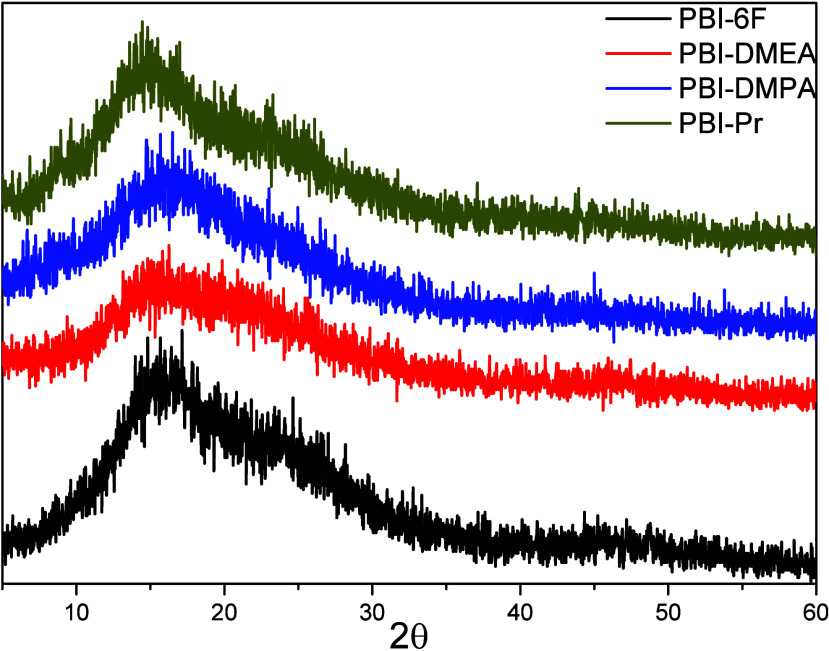
WAXD spectra of pristine PBI-6F and N-modified PBIs.

A 7–9% decrease in the densities was detected after N-substitution. This may be a consequence of two simultaneous effects: further separation of polymer chains due to elimination of the hydrogen bonding and a decrease of relative content of the heavy F atoms per repeating unit because of the introduction of the substituents. The intermolecular chain distance roughly estimated by values of *d*-spacing also increased for N-substituted PBIs, and the biggest *d*-spacing was noted for PBI-Pr, which coincided with its lowest density. The FFVs of the polymers calculated based on their density also showed higher FFV values than those of the original PBI-6F, although the differences were insignificant. This can be explained by the presence of bulky CF_3_ groups in the main chain of the polymers, which further restricted torsional mobility of the polymer chains, causing the loose packing. The introduction of substituents at the imidazole N atom in these PBIs did not result in a significant increase in FFV, as the substituents are plausibly able to accommodate themselves in the empty space of the polymer matrix. A similar tendency in FFV was observed in the cases of other N-substituted PBIs with bulky groups in the main chains.^17^ Interestingly, alkyl substituents with terminal dimethylamine groups did not cause an additional increase in the FFV of the polymers compared to the pure alkyl substituent, as can be seen in Table 1. Despite the increase in the van der Waals volume of the dimethylamine group, these remained very flat within the polymer and, therefore, did not produce bigger FFV. Alkylamine with a longer chain occupied more free space within the polymer matrix, which resulted in some decrease of FFV (compare data for PBI-DMPA and PBI-DMEA in Table 1). Same dependence of side chain length on FFV was observed for rigid poly­(norbornene) backbones modified with flexible aliphatic groups.^31^

Self-supporting dense films from all PBIs synthesized in the study with a thickness of 40–50 μm were tested for pure gas permeation. The membranes formed were dried at 200 °C in a vacuum for 10 h; however, as discussed above, not all residual liquids were removed under these conditions; so additional heat treatment at 300 °C was required to obtain completely dry membranes without any low molecular weight compounds. Pure gas permeability and selectivity coefficients for the membranes measured at 3 bar and 35 °C after heat treatment at 200 and 300 °C are given in Table 2, and for CO_2_/CH_4_ gas pair plotted in comparison with Robeson’s upper bound in Figure 7. As can be seen permeabilities of the membranes after drying at 200 °C were low, lying far below the 1991 upper bound. The modification of NH with DMEA, DMPA, and Pr chains produced membranes with higher permeability, therefore, suggesting that the introduction of the aliphatic chain at imidazole N provided steric hindrance, increasing polymer chain separation and fractional free volume. However, the modified membranes demonstrated reduced selectivity that did not allow us to locate them any closer to the upper-bound line. Nevertheless, as shown by thermal analysis, these membranes still contained some residual solvents that may affect their gas separation performance. And, indeed, after heating at 300 °C, when all residual low molecular weight compounds were removed completely, the membranes displayed significant enhancement in gas transport properties; their permeabilities increased two or three times with respect to all gases (Table 2). Solvents or any other low molecular weight contaminants present in the polymers can act as plasticizers, increasing chain mobility and gas solubility and, thus, improving gas permeability through the membrane. However, it was found that the amount of residual solvent in the membranes studied after treatment at 200 °C was too insignificant (below 2% by weight) to cause a plasticization phenomenon. On the contrary, the effect of low concentrations of additives in the polymer matrix can have an antiplasticizing effect and significantly reduce gas permeability due to the reduction in the free volume of the polymer and competitive sorption at Langmuir sites.^32–34^ In fact, density measurements of the membranes showed a notable decrease of almost 20% after treatment at 300 °C, for PBI-DMEA and PBI-DMPA, while decrease in densities for PBI-Pr and PBI-6F was smaller 11% and 6% respectively. This resulted in the corresponding increase in their FFV and, thus, in improved permeabilities. Importantly, the selectivity indexes also increased or at least stayed practically unchanged, allowing their total permselectivity to get closer to the upper line (Figure 7). Such behavior is characteristic of rigid-chain polymers after thermal treatments, when an increase in free volume accompanies a narrowing of the size distribution of the free volume elements, thereby preserving or enhancing the membrane’s size-sieving ability.^35,36^ Once again, the permeabilities of N-modified PBIs for practically all gases were higher than those of the original PBI-6F, which generally coincided with the higher FFVs of the modified polymers. However, no direct correlation was found between the FFV and permeability within the N-modified PBIs. The FFV values of N-substituted PBIs were very similar, but their permeabilities differed significantly. The lowest permeability among the substituted PBIs was detected for PBI-Pr with a pure alkyl substituent, while both membranes modified with tertiary amine substituents, PBI-DMEA and PBI-DMPA, demonstrated much higher permeabilities. The permeability of PBI-Pr was close to the values of unmodified PBI-6F for the majority of gases, but it was significantly less selective (Table 1). On the other hand, gas transport properties of PBI-DMEA and PBI-DMPA were particularly improved with respect to the CO_2_/CH_4_ gas pair (Figure 7). Thus, for the CO_2_ permeability of PBI-DMPA a more than 3-fold increase was observed in comparison with that of raw PBI-6F, both having close values of selectivity. A very similar trend was noted for the PBI-DMEA membrane. This resulted in PBI-DMEA and PBI-DMPA surpassing the 1991 upper bound for CO_2_/CH_4_. This suggested that the increase in permeability noted for the studied systems was not directly related to a more open polymer matrix in accord with previously reported data for aromatic PBI.^8,15^ Except for FFV, other factors, such as the *T*_g_ of the polymer or gas–polymer interaction, influence polymer gas transport characteristics. Due to the relatively low thermal stability of the N-modified PBIs, it was difficult to determine their *T*_g_. However, analyzing the DSC curves we did not see anything that may be interpreted as phase transitions until 310–330 °C, depending on the polymer. So, the heat treatment of the polymers at 300 °C seems to be below their *T*_g_’s. It was reported that N-substitution of PBIs with alkyl groups led to a general decrease in gas sorption,^15^ which may be a reason for a very moderate improvement in permeability for PBI-Pr as compared to that for PBI-6F. Meanwhile the presence of terminal *tert-*amino moieties may, on the contrary, increase the sorption and/or solubility. In attempts to clarify the effects, we calculated the solubility and diffusivity parameters for the membranes, which are displayed in Table 3. The bulkiness of the substituent increases the average distance between chains, as can be seen from WAXD data, therefore enhancing the diffusion coefficient (*D*) for all the polymers. An important finding is that the drying procedure at 300 °C dramatically improves the gas separation performance, especially for the membranes bearing N-substituents with tertiary amine groups (PBI-DMEA and PBI-DMPA). Elimination of all residual low molecular weight compounds from the membrane resulted in enhancing the available FFV and increasing the *D* coefficients in all cases. Improvement in diffusivity was the biggest for the parent PBI-6F; its diffusivity coefficients doubled after treatment at 300 °C. Meanwhile, an increase in diffusivity was lesser for the N-substituted PBIs. Thus, the increase in diffusivity did not explain the important improvement in gas separation observed for the DMEA and DMPA polymers. However, the solubility coefficients demonstrated significant growth for the polymers with amine functionalization in the aliphatic chain (PBI-DMEA and PBI-DMPA). As seen from Table 3, the solubility coefficients also increased for all polymers after the additional heat treatment, but for PBI-DMEA and PBI-DMPA the increase was larger. Thus, the overall CO_2_/CH_4_ solubility selectivity increased more than two times for PBI-DMEA 300 and PBI-DMPA 300, together with the overall highest CO_2_ permeability (135.4 Barrer), allowing PBI-DMPA 300 to overcome the 1991 upper limit. The amine functionalization should enhance the specific chemical interaction with the quadrupole moment of CO_2_ increasing the solubility coefficient and effectively tuning the balance between the kinetic component (*D*) and the thermodynamic component (*S*). While the *N*-alkylamine substitution successfully enhances the CO_2_/CH_4_ separation properties of the membranes, the observed reduction in thermal stability restricts their use in high-temperature processes such as syngas or hydrogen separation. Importantly, this thermal limitation does not affect other industrial applications like postcombustion flue gas and biogas upgrading, where operating temperatures are typically moderate, falling well below 100 °C. In the moderate-temperature window, these N-PBI systems obtained by a simple and inexpensive method offer a competitive advantage over conventional commercial glassy polymers, such as cellulose acetate, polysulfone, and various polyimides.^37,38^

**Table 2:** Ideal Permeabilities and Selectivities of the PBI Membranes Studied in This Work

	**Permeability**					**Selectivity**	
**Polymer** ^a^	**He**	**N**_**2**_	**O**_**2**_	**CH**_**4**_	**CO**_**2**_	**O**_**2**_ **/N**_**2**_	**CO**_**2**_ **/CH**_**4**_
**PBI-6F 200**	41.9	0.52	3.06	0.40	13.61	5.9	34.0
**PBI-6F 300**	100.2	1.65	9.39	1.04	42.09	5.7	40.5
**PBI-DMEA 200**	44.4	1.21	5.83	1.15	30.58	4.8	26.6
**PBI-DMEA 300**	94.9	4.06	21.25	3.39	109.7	5.2	32.4
**PBI-DMPA 200**	43.1	1.50	7.21	0.98	32.5	4.8	33.2
**PBI-DMPA 300**	106.2	5.07	25.19	3.87	135.4	5.0	34.9
**PBI-Pr 200**	57.0	1.45	6.71	1.85	37.23	4.6	20.1
**PBI-Pr 300**	112.4	2.19	10.35	2.90	57.22	4.7	19.7

a 200 and 300 correspond to PBIs after treatment at 200 and 300 °C respectively.

**Table 3:** Apparent Diffusion (*D*) and Solubility (*S*) Coefficients of Membranes and Their Selectivities

	***D*** **(10** ^**–8**^ **cm** ^**2**^ **/s)**			***S* (10^**–2**^ cm^**3**^(STP)/** **cm** ^**3**^ **cm Hg)**		
**Polymer**	***D***_***CH4***_	***D***_***CO2***_	***D***_***CO2***_ **/** ***D***_***CH4***_	***S***_***CH4***_	***S***_***CO2***_	***S***_***CO2***_ ***/S***_***CH4***_
**PBI-6F 200**	0.104	1.24	11.90	3.40	11.5	3.37
**PBI-6F 300**	0.207	2.74	13.23	4.45	15.4	3.45
**PBI-DMEA 200**	0.631	2.87	4.53	1.72	10.6	6.15
**PBI-DMEA 300**	0.668	3.46	5.18	3.15	28.6	9.08
**PBI-DMPA 200**	0.386	1.45	3.76	1.42	5.70	4.03
**PBI-DMPA 300**	0.494	2.73	5.53	3.19	30.8	9.65
**PBI-Pr 200**	0.399	1.64	4.10	2.15	9.88	4.64
**PBI-Pr 300**	0.523	2.43	4.64	3.02	14.20	4.70

**Figure 7:**
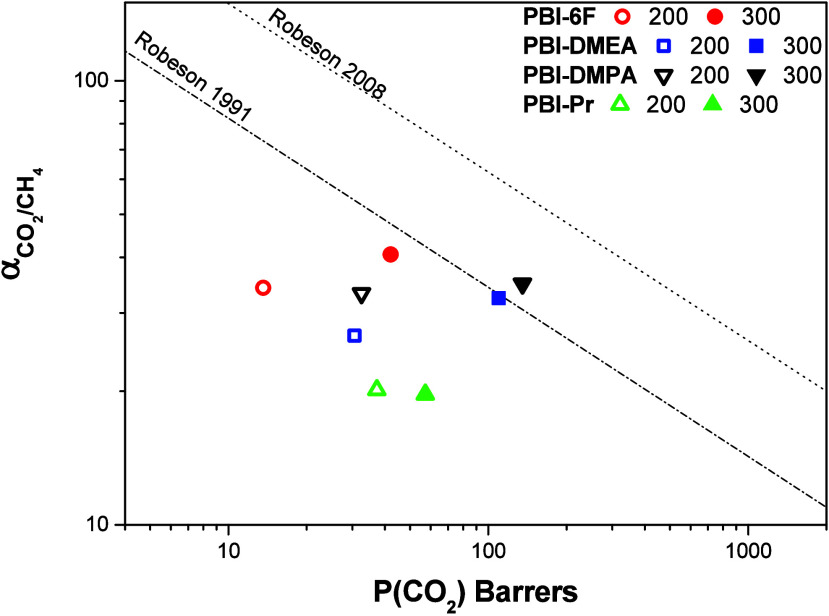
Permeability vs selectivity plot for the CO_2_/CH_4_ gas pair.

## 4 Conclusions

Post-polymerization N-substitution of PBI-6F by alkyl and alkyl amine groups was successfully carried out using a simple synthetic procedure in 90% yields. Elimination of hydrogen bonding and chemical structure of N-substituted PBIs was confirmed by ^1^H NMR and FT-IR spectral analyses. N-Modified polymers were soluble even in ordinary organic solvents such as THF and CHCl_3_. To measure gas transport properties, dense membranes of good qualities were prepared by solution casting. However, despite the use of a solvent such as CHCl_3_, it was very difficult to remove the solvent from the membranes. In order to obtain completely dry membranes, without any residual liquid left, a multistep heating treatment was performed. It was demonstrated that permeation and gas-separation behavior of the membranes were affected to great extent by the presence of even residual traces of solvent. All N-substituted membranes demonstrated higher permeability values in comparison with pristine PBI-6F, which is associated with increasing polymer chain separations due to the absence of hydrogen bonding and the presence of bulkier substitutents. However, permselectivity was particularly improved for membranes with alkylamine chains at the N-position, PBI-DMEA and PBI-DMPA, especially with respect to the CO_2_/CH_4_ gas pair, which resulted in these membranes surpassing the 1991 upper bound. The N-substituted membrane with a pure alkyl group, PBI-Pr, exhibited the lowest increase in permeability among all modified membranes despite its highest FFV. This behavior was attributed to the increase in the solubility parameter of the alkyl amine substituted PBIs due to the interaction of the amine moiety with the polar CO_2_.

TGA analysis demonstrated the complex character of decomposition of PBI-DMEA and PBI-DMPA with the first step starting just above 300 °C. Theoretical calculations performed unexpectedly showed that the weakest bond in these membranes was the C–C bond next to the amine group in the substituent but not the C–N bond attached to imidazole as generally accepted. The post-polymerization modification of PBI-6F with N-alkylamine groups proved to be a simple and effective method for enhancing CO_2_/CH_4_ separation performance, surpassing the 1991 Robeson upper bound, placing this polymer above some commercial polymers. Although TGA analysis demonstrated a reduced thermostability, these materials may be a good option for moderate-temperature applications, such as postcombustion CO_2_ capture and biogas upgrading, where the operational window is typically below 100 °C. However, further studies on gas mixtures and plasticization are needed to clarify the scope of this modification.
